# Determining EDC exposure: direct analytical methods are essential for accurate analysis of human biospecimens

**DOI:** 10.1210/jendso/bvag076

**Published:** 2026-03-31

**Authors:** Roy Gerona, Alia Sovereign, Deborah French, Mikayla Joyce Gonzaga, Frederick S vom Saal, Patricia Hunt

**Affiliations:** Department of Obstetrics, Gynecology, and Reproductive Sciences, University of California, San Francisco, CA 94134, USA; Department of Obstetrics, Gynecology, and Reproductive Sciences, University of California, San Francisco, CA 94134, USA; Departments of Laboratory Medicine, University of California, San Francisco, CA 94107, USA; Department of Obstetrics, Gynecology, and Reproductive Sciences, University of California, San Francisco, CA 94134, USA; Division of Biological Sciences, University of Missouri-Columbia, Columbia, MO 65211, USA; School of Molecular Biosciences and Center for Reproductive Biology, Washington State University, Pullman, WA 99164, USA

**Keywords:** endocrine disrupting chemicals (EDCs), analytical chemistry, biomonitoring, exposure, risk assessment

## Abstract

**Purpose:**

Estimates of human exposure are a major component of chemical risk assessment. Studies of bisphenol A (BPA) have raised concern that exposure has been underestimated because the lack of standards for the measurement of the major BPA metabolites has necessitated the use of flawed analytical tools to indirectly estimate them. Because other endocrine-disrupting chemicals (EDCs) are measured using similar indirect methods, we evaluated the accuracy of indirect analysis for representatives from three different classes of non-persistent EDCs that undergo rapid phase II metabolism: bisphenols, parabens, and phthalates.

**Methods:**

A direct LC–MS/MS method that simultaneously measures bisphenol S (BPS), propyl paraben (PrP), and monobutyl phthalate (MBP), and their major metabolites in urine, was used to quantify these EDCs in sixty second-trimester human urine samples. The same samples were also analyzed with a widely used indirect method that requires enzymatic hydrolysis before estimating metabolite levels.

**Results:**

Marked discrepancies were evident when maternal urine samples were analyzed by both direct and indirect methods. Indirect analysis underestimated levels of all three EDCs and BPA, with the magnitude of underestimation varying by analyte.

**Conclusion:**

The accuracy of widely used “indirect” analytical methods that estimate metabolite levels in human urine is neither predictable nor consistent. Greater precision and accuracy is attained using authentic standards for metabolites. Given the importance of biomonitoring data in estimating human EDC exposure, analytical accuracy is critical. Availability of standards for both the parent compound and its major metabolites should be required *before* a chemical enters the marketplace.

Exposure to synthetic endocrine disrupting chemicals (EDCs), chemicals that can mimic or interfere with the actions of endogenous hormones, is a ubiquitous part of 21st century life. During the past several decades, the levels and spectrum of EDC contaminants in air, water, soil, food, and consumer products have increased rapidly. The most common contaminants and their associated health concerns have been recently summarized by the Endocrine Society and in recent reviews (eg, [1-3]).

The impact of exposure to EDCs is particularly profound during sensitive developmental windows, ie, the fetal period, early postnatal life, and childhood. Experimental studies in multiple species provide evidence of adverse exposure effects on the developing brain, heart, lungs, reproductive organs, and immune system that are correlated with behavioral changes, metabolic diseases like diabetes and obesity, increased risk of cancer, and reduced fertility (reviewed in [4-7]). Because measuring levels of EDCs in the human fetus is not feasible, maternal biospecimens have been used to estimate fetal exposure. Maternal urine is preferred for biomonitoring because it is easy to obtain and generally considered to better capture exposure than the transient levels of rapidly metabolized, non-persistent EDCs present in circulating blood. Thus, we focused our analyses on EDC levels in maternal urine from early second-trimester pregnancies.

Rapid expansion of EDC chemicals has been driven both by the inclusion of new members to enhance performance and to replace chemicals whose biological and/or environmental effects have raised concern. Bisphenol A (BPA) and replacement bisphenols provide a striking example of the latter: As evidence of adverse effects induced by BPA exposure accumulated and regulatory restrictions were imposed, manufacturers moved to replace BPA with other bisphenols in some products. The rapid emergence of these “BPA-free” substitutes did not decrease harmful EDC exposure, since substitutes like bisphenol S (BPS) also appear to pose significant health risks (eg, [8, 9]). Importantly, despite the strong and ever-growing evidence of adverse effects induced by BPA exposure, the decision by the European Food Safety Authority to dramatically lower the tolerable daily intake for BPA has met with considerable resistance from other regulatory agencies in England, Germany, and the USA [10].

Understanding the hazards ascribed to an individual chemical and the doses at which effects are elicited in different tissues is essential in assessing the risk it poses to human health. Critically, in addition to identifying and characterizing the hazards posed by exposure during vulnerable periods of development and in adulthood, risk assessment requires accurate estimates of levels of human exposure. In chemical risk assessments, risk = hazard × exposure, and if exposure is deemed minimal, risk is deemed minimal regardless of the intrinsic hazard posed by the chemical [11]. This approach is considered unsound by adherents to the principle of sustainable chemistry who argue that intrinsically hazardous chemicals should not be used in products whose use would lead to widespread exposure [12].

The U.S. Environmental Protection Agency (EPA) guidelines for chemical exposure assessment outline three main approaches for assessing levels and routes of human exposure: (1) “direct”—point of contact measurements to assess levels of chemicals taken into the body through inhalation, ingestion, or dermal uptake, (2) “exposure reconstruction”—exposure estimates based on levels measured in human tissues and body fluids, and (3) “indirect” assessments—exposure scenarios that often use a set of assumptions based on models to obtain estimates of humans exposure and pharmacokinetics. These models typically do not consider the possibility that the chemical has hormonal or antihormonal activity and can interfere with the activity of endogenous hormones. Because they can exert effects at extremely low exposure levels—levels below those that can be measured—the prediction that there is a threshold (safe) exposure level for EDCs has been disputed [13, 14].

Most of our understanding of human EDC exposure is based on exposure reconstructions using data from biomonitoring studies of human populations. The most widely used biomonitoring data are from the National Health and Nutrition Examination Survey (NHANES). NHANES is a set of ongoing surveys conducted by the Centers for Disease Control and Prevention (CDC) that combines data from interviews and physical examinations of about 5000 individuals per year to obtain health statistics for the U.S. The program has included the collection of biospecimens (serum, plasma, urine, and DNA) for measurement of EDCs since 1999. Over the past twenty-five years, these biospecimens have provided insight to levels of common environmental EDC contaminants like BPA in our bodies (Table 1), but there are estimated to be over a thousand [15].

**Table 1:** Geometric mean (95% CI) or urine concentration (mcg/g creatine) in women: NHANES 1999 to 2016

	99-00	01-02	03-04	05-06	07-08	09-10	11-12	13-14	15-16	17-18
Bisphenol A			2.8	2.0	2.4	2.1	1.9	1.4	1.2	
			(2.50–3.08)	(1.95–2.14)	(2.17–2.57)	(1.92–2.27)	(1.76–2.08)	(1.23–1.51)	(1.11–1.29)	
Bisphenol S								0.48	0.53	
								(0.43–0.53)	(0.45–0.58)	
Propyl paraben				23.9	22.0	20.4	16.0	14.4	10.3	
				(19.9–28.8)	(18.7–25.8)	(17.8–23.3)	(13.9–18.4)	(12.1–17.2)	(7.96–13.4)	
Mono-n-butyl phthalate	28.6	21.7	24.8	22.9	23.1	17.8	9.8	10.4	11.7	10.2
	(25.3–32.3)	(19.6–23.9)	(22.9–26.8)	(20.9–25.1)	(21.0–25.5)	(16.0–19.7)	(8.86–10.9)	(9.8–11.1)	(10.8–12.6)	(9.26–11.2)

n > 1000 for each reported value.

Because analytical data are released biennially, NHANES data also provide a means of assessing temporal changes in exposure. For BPA, NHANES data provide evidence of decreasing levels in human urine coincident with the emergence of replacement bisphenols (eg, [16] and Table 1). For example, levels of BPS in urine were reported in a 2012 study to vary among countries, with the highest geometric mean concentration (0.933 µg/g creatine) reported in urine samples from Japan, reflecting its use as a primary replacement for BPA in food-contact materials [17].

Conclusions about human exposure based on trends in NHANES data assume accuracy in the data and our extrapolations of it. But the apparent decreases in urine levels of BPA, mono-n-butyl phthalate and n-propyl paraben in the US population in data reported by the CDC in the last two decades (Table 1) are at odds with the steady increases in annual production and the myriad uses of BPA [18, 19], dibutyl phthalate [20, 21] (the precursor of bioactive mono-n-butyl phthalate) and n-propyl paraben [18, 22, 23].

Non-persistent EDCs like BPA, parabens, and phthalates are rapidly metabolized, and because of this, are present in urine predominantly as polar (conjugated) metabolites. BPA, for example, undergoes Phase II metabolism in the liver, where it is primarily glucuronidated or, less frequently, sulfated [24]. The metabolites are hydrophilic, readily transported in blood, and preferentially eliminated by the kidneys. Thus, determining levels in urine requires accurate measurement of both the parent BPA molecule and its major polar metabolites. The same is true for mono-n-butyl phthalate, BPS, and n-propyl paraben, which, although more hydrophilic than BPA, are also conjugated (glucuronidated) in the liver before elimination by the kidneys.

Readily available standards for parent compounds, like BPA, have allowed them to be measured directly. The lack of comparable standards for metabolites, however, has necessitated the use of indirect methods to estimate levels in urine. These methods operate on the assumption that metabolites of different parent EDCs can be enzymatically deconjugated, transforming them back to the parent compound, which can then be measured. The method is “indirect” because levels of parent compound (eg, BPA) measured in urine samples pre- and post-deconjugation are used to infer conjugated metabolite levels. In the absence of authentic standards for metabolites like BPA glucuronide (BPA-G), the reaction kinetics have been monitored using a surrogate chemical with an available standard (for BPA glucuronide, it is 4-methylumbelliferone glucuronide), and three assumptions have been made:

1. The deconjugation reaction (BPA-G → BPA) is complete or nearly so (ie, all BPA-G is deconjugated).
2. The end result of enzymatic deconjugation is the parent compound, BPA.
3. Deconjugation of the surrogate (4-methylumbelliferone-glucuronide to 4-methylumbelliferone) accurately predicts deconjugation of BPA-G to parent BPA.

When authentic standards for the major metabolites of BPA (BPA-G and BPA-S) became available, we developed a direct method to simultaneously analyze BPA and both metabolites [25]. Surprisingly, in some urine samples measured using both indirect and direct methods, indirect analysis significantly underestimated the major metabolite, BPA-G. Subsequent analyses suggested both that deconjugation procedures were incomplete and that enzymatic deconjugation of BPA-G was not yielding the expected product, unconjugated parent BPA. Further, the inaccuracy of the method was exacerbated with increasing levels of BPA, and samples with the highest levels of BPA-G showed the most dramatic underestimation via indirect analysis [26]. We interpreted this as evidence that BPA levels likely were markedly underestimated in biomonitoring studies using indirect analytical methods. Our findings met with considerable resistance (eg, [27, 28]) and insistence that the indirect methodology had been validated, providing assurance of accuracy [27]. This argument, however, conflates *replication* with *accuracy*, ie, reproducibility of results provides no assurance of the ability of the method to accurately capture levels of total BPA in biospecimens.

The public health implications are substantial: Since accuracy was inversely related to the concentration of BPA in the sample, our findings suggest flawed analytical tools have resulted in significant underestimation of the range of human exposure. Indeed, as we were able to demonstrate using our data, this would cause compression of exposure quartiles often used in epidemiological studies, reducing the power to detect exposure effects [29]. The findings also raise concern that the problem may extend to other phenolic compounds measured using similar indirect analytical methods [26]. The present study was undertaken to assess the accuracy of indirect methods for a replacement bisphenol and two other EDCs. The response to our findings with respect to BPA prompted us to design the current investigation as a collaborative study with two independent laboratories analyzing replicate samples. In addition to BPA and its metabolites (BPA-G and BPA-S), we included in our analyses bisphenol S (BPS), BPS glucuronide (BPS-G), and BPS sulfate (BPS-S), monobutyl phthalate (MBP) and MBP glucuronide (MBP-G), propyl paraben (PrP) and PrP glucuronide (PrP-G). Authentic standards for MBP-sulfate and PrP-sulfate were not available.

We report here results suggesting considerable variation among these EDCs in the accuracy of measured metabolite levels obtained using indirect analytical methods, with striking differences, even between the structurally related chemicals BPA and BPS, demonstrating that pharmacology is more important than structural similarity. We further address methodological complexities encountered during our analyses that underscore the importance of careful monitoring for contaminants as well as for the quality and performance reproducibility of standards, reagents, and laboratory supplies.

## Materials and methods

### Chemicals and reagents

Standards for the parent compound and major metabolites of Bisphenol S, and monobutyl phthalate glucuronide, Bisphenol S^13^C_12_, propyl paraben −^13^C_6_, and BPS glucuronide-^13^C_12_ were obtained from Toronto Research Chemicals (North York, ON, Canada). Monobutyl phthalate was obtained from Sigma Aldrich (St. Louis, MO), and monobutyl phthalate-d4 and propyl paraben glucuronide were obtained from Santa Cruz Biotechnology (Dallas, TX). Propyl paraben was obtained from Cayman Chemical (Ann Arbor, MI). Additional chemicals and reagents used included acetic acid and β-Glucuronidase from Helix pomatia Type H-3 from Sigma Aldrich (St. Louis, MO), HPLC-grade water and HPLC-grade ammonium acetate from Fisher Scientific (Waltham, MA), B&J LC-MS grade Methanol from VWR International (Radnor, PA), and synthetic human urine from UTAK Laboratories (Valencia, CA).

### Human urine specimens

Urine samples were from a study cohort of pregnant women between 18 and 45 years of age in the second trimester (13–24 weeks) of pregnancy [25]. Maternal/fetal samples were collected from over 800 second-trimester pregnancies at the Women's Option Center at Zuckerberg San Francisco General Hospital between 2008 and 2017 under NIH project R01 ES013579. Patient consent and collection of maternal and fetal biospecimens by the clinic was conducted under approved UCSF IRB 10-02246 and was fully compliant with federal requirements for research using human fetal tissues. Urine samples were aliquoted into 5 mL polypropylene cryovials at the time of collection and stored at −80 °C until analysis.

### Indirect and direct analytical methods

The development and validation of a direct method for simultaneous analysis of bisphenol S, propyl paraben, monobutyl phthalate, and their metabolites is summarized in Supplemental Methods [30]. Using commercially available standards, method development involved both the blinded evaluation of samples of synthetic urine spiked with known amounts of each chemical metabolite and comparative analysis of 60 human urine samples analyzed using both direct and indirect methods. Sample preparation and analysis for the current studies followed the methods detailed therein.

Samples were run on an Agilent 1260 HPLC coupled to an AB Sciex 5500 triple quadrupole mass spectrometer (LC-MS/MS) with electrospray ionization in negative mode using a Zorbax Extend-C18 column (4.6 × 100 mm, 1.8 µm; Agilent Technologies). Isotope dilution was applied for quantitative analysis using the isotopologue of each analyte as an internal standard. A summary of the development of our direct method and the internal standard for each analyte is provided in the Supplemental Methods [30]. Data analysis was performed using AB Sciex Analyst and MultiQuant software.

### Quality assessment and control

All collection and processing materials were evaluated for contamination, and reference blanks were included in each run. To monitor for contamination in the instrument and sample preparation, each batch run included both a solvent-only blank and a matrix-only blank. In addition, to assess performance, two quality control samples at low and high concentrations that spanned the linear dynamic range of each analyte were included in each run. For acceptance, the results of a batch run had to meet the acceptance criteria for both the relative error (<15%) and the imprecision of quality control samples (CV <15%) based on US FDA method validation guidelines [31]. The relative error was calculated as the difference between the measured value and the spiked value divided by the spiked value. The coefficient of variation was calculated as the standard deviation divided by the mean of three replicates and presented as a percent.

Prior to use, each new lot of a standard and critical supplies was evaluated for suitability: For standards, the concentration of a new lot was verified by comparison with the existing lot; a relative error of ≤15% was required for acceptance. For supplies (eg, solid-phase extraction cartridges), calibration curves for all analytes were prepared using the new lot. For approval for use, a linear regression coefficient of at least 0.95 was required. In addition, low- and high-concentration QC samples prepared with the new lot were evaluated using a calibration curve generated with the previous lot. Approval for use required a relative error ≤ 15% and a coefficient of variation ≤ 15%.

### Proficiency testing

Two laboratories at different institutions were provided six proficiency testing samples containing compounds spiked into synthetic human urine (UTAK Laboratories, Valencia, CA) as shown in Table S1 [30]. Samples of synthetic human urine spiked with different levels of parent compounds and metabolites were prepared by a third laboratory and provided to both laboratories as blinded samples for analysis. In addition, each lab received seven individual analyte standards (10 mcg/mL in methanol), four internal standards (10 mcg/mL in methanol), and 5 mL of synthetic drug-free urine. As required for external proficiency testing, each laboratory submitted average values obtained for each proficiency testing sample to a third party. For each measurement, the relative error was calculated, and the mean and standard deviation of the relative error were determined for each chemical. After results were submitted from both labs, contents and concentrations of each sample, as shown in Table S1 [30] were provided.

### Direct and indirect method comparison

Sixty second-trimester maternal urine samples were analyzed using both direct and indirect methods across four analytical batches. Each sample underwent concurrent analysis by both methods. For comparison of results obtained by the two methods, the total concentration of each parent chemical and its metabolites was compared to determine the correlation between the results from the two methods. For further comparison, the measurements obtained from the direct method were ranked from lowest to highest. Ranked samples were then subdivided into four quartiles (*n* = 15 per quartile), and for each quartile, the geometric mean concentration was calculated. Corresponding measurements obtained by indirect analysis were compared, geometric means calculated, and the fold change between measurements determined. Measurements below the LOD were imputed as LOD divided by the square root of two for the geometric mean calculations.

## Results

### Development, validation, and proficiency testing of direct methods

Samples of synthetic human urine spiked with different levels of parent compounds and metabolites were prepared by a third laboratory and provided to both laboratories as blinded samples for analysis. Because standardized performance guidelines for environmental biomonitoring are lacking, the acceptance limits for proficiency testing were based on existing regulatory and proficiency testing frameworks, including the Clinical Laboratory Improvement Amendments (CLIA) [31], a US federal program that sets quality standards for all laboratory testing on human samples, and national external quality assessment programs that define allowable total error as a percentage around the target value, commonly in the range of 10–25%. We adjusted the acceptance criterion to 25% to be more lenient toward method variability.

**Figure 1:**
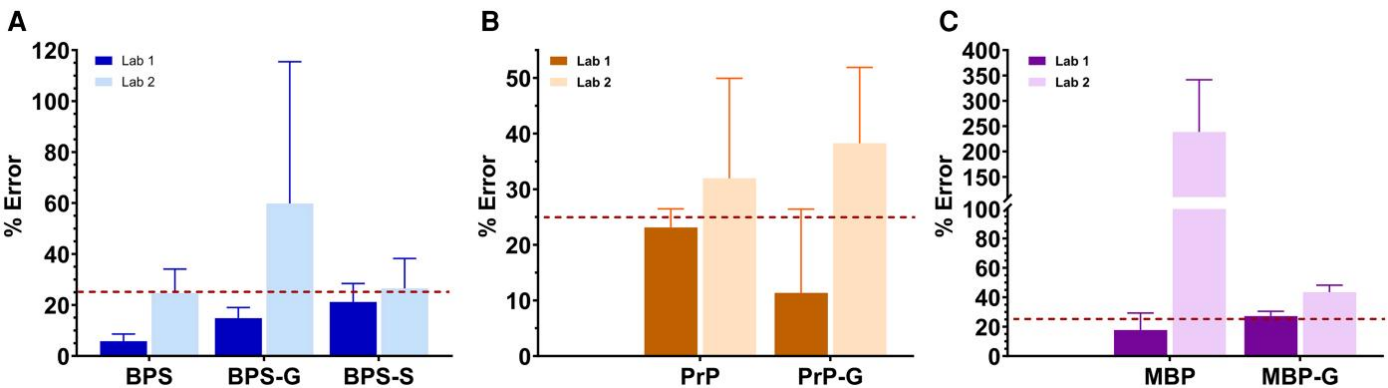
Proficiency testing results for direct analysis. Percent errors (mean ± standard deviation) for measurements reported by the two laboratories for bisphenol S (A), propylparaben (B), and monobutyl phthalate (C). Dark and light bars denote Laboratory 1 and 2, respectively, and the horizontal line indicates the 25% relative-error acceptance threshold. On the basis of these data, Laboratory 2 was eliminated from the study.

Two rounds of testing demonstrated accuracy for one of the two labs provided with samples (Fig. 1). With the exception of one chemical, whose mean relative error was 27%, measurements submitted by Lab A had mean relative errors below 25%. In contrast, for Lab B, the relative error for all chemicals except BPS exceeded 25%. Hence, Lab B withdrew, and all subsequent results are derived *only* from Lab A, the laboratory able to demonstrate proficiency.

### Comparison of analytical methods

To assess the ability of currently used methods for the three chemicals to accurately capture levels of metabolites, we compared results obtained when urine specimens were analyzed using both an “indirect” method to infer levels of metabolites and our “direct” method. BPA was included as a positive control. Sixty urine samples from our cohort of maternal biospecimens from second-trimester pregnancies were analyzed. The linear range of concentrations for the three EDCs of interest (BPS, MBP, and PrP) and BPA measured by both methods is summarized in Table S2 [30]. For the direct method, total levels were calculated by converting each conjugated metabolite to its equivalent amount of the unconjugated parent compound and then adding these values to the measured unconjugated concentration. For the indirect method, the post-deconjugation measurement was used as the total level.

**Figure 2:**
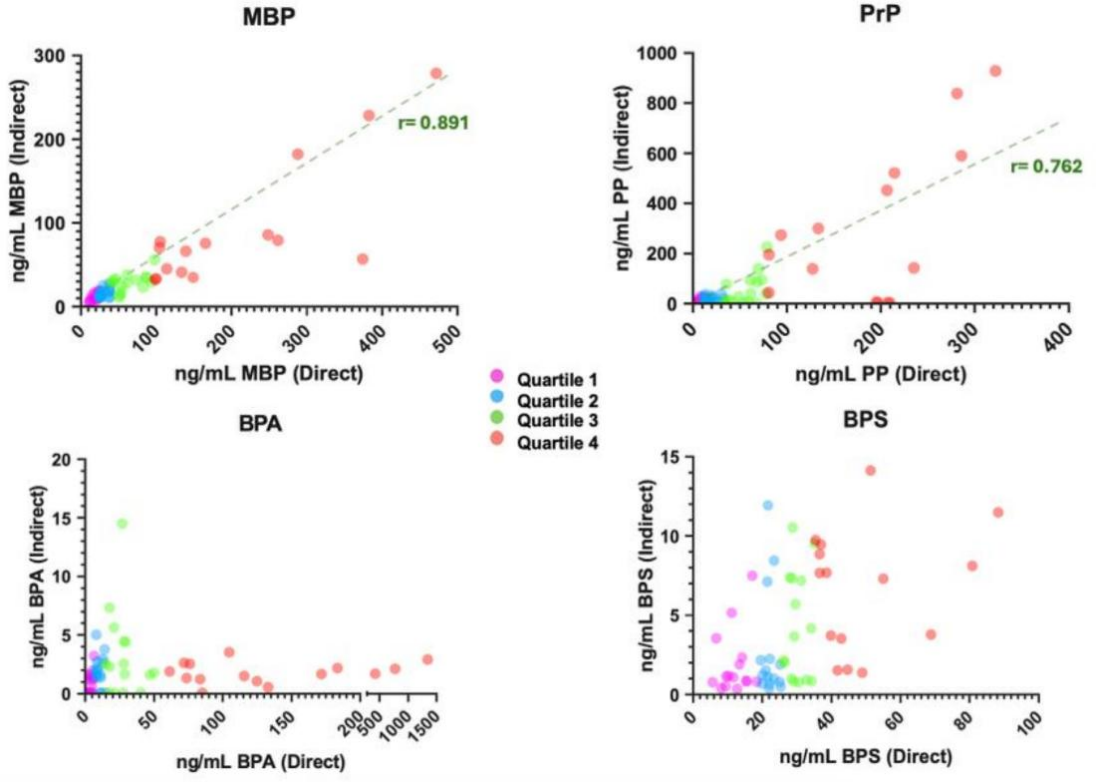
Comparison of results for sixty maternal urine samples analyzed by direct and indirect methods. Correlation between total concentrations measured by direct (*x*-axis) and indirect (*y*-axis) analysis for MBP, PrP, BPA, and BPS. Dashed line denotes best-fit linear regression, with corresponding regression coefficient for MBP and Prp, with linear profiles. Colors denote samples in quartile 1 (fusia), 2 (blue), 3 (green), and 4 (orange) based on concentrations obtained via direct-analysis. Note that, due to disparities between results obtained by the two methods, relative ranges for the *x*- and *y*-axes differ markedly for BPA and BPS.

Although indirect analysis underestimated levels of all three EDCs, there were marked differences among analytes. Evaluation of the agreement between results obtained by direct and indirect analysis demonstrated a linear relationship for both MBP and PrP, with correlation coefficients of 0.891 and 0.762, respectively (Fig. 2). For BPA, as in our previous study, the magnitude of the underestimation increased in a concentration-dependent manner. In contrast, BPS was also underestimated by the indirect method, but the magnitude of underestimation was similar for all concentrations.

To further analyze the effect of concentration and the degree to which indirect analysis under- or overestimated levels, samples were stratified into quartiles on the basis of direct measurements (Fig. 2). Stratification provided a quantitative assessment of the accuracy of results obtained by indirect analysis. Geometric means calculated for each quartile were used to calculate the fold-change between direct and indirect measurements and assess the effect of concentration (Table 2; Fig. 2). A significant increase in fold-change across quartiles confirmed the strong concentration dependence of BPA underestimation, with quartile 1 showing a 4.4-fold underestimation by the indirect method while quartile 4 showed a 94.4-fold underestimation of the indirect relative to the direct method (Table 2; Fig. 2).

**Table 2:** Comparison of geometric means from DIRECT and INDIRECT analysis*^a^*

		Quartile 1	Quartile 2	Quartile 3	Quartile 4
**BPA**	Direct	3.5	10.3	25.9	151.0
	Indirect	0.8	1.5	1.9	1.6
	Fold difference	4.4×	6.9×	13.6×	94.3×
**BPS**	Direct	11.3	22.2	29.7	47.6
	Indirect	1.2	1.6	2.6	5.3
	Fold difference	9.4×	13.9×	11.4×	9.0×
**MBP**	Direct	18.5	31.2	63.1	180.7
	Indirect	10.6	15.7	27.1	72.9
	Fold difference	1.7×	2.0×	2.3×	2.5×
**PrP**	Direct	4.7	19.3	55.7	175.6
	Indirect	2.9	14.2	23.2	86.6
	Fold difference	1.6×	1.4×	2.4×	2.0×

^*a*^Levels of each chemical obtained by direct analysis of 60 urine samples were used to separate data into quartiles. Numbers indicate fold underestimation of indirect analysis (ie, a comparison of geometric means).

Although BPS was also consistently underestimated by the indirect method, its fold-change remained relatively constant (∼10×) across quartiles. In contrast, MBP showed a gradual increase in fold-change with concentration (1.7–2.5×). For PrP, the fold-change also was modest (1.4–2.4×), but notably, indirect analysis also resulted in overestimation for some samples (Fig. 2). The results for PrP were particularly interesting; despite a linear correlation between results obtained by the two methods, indirect analysis showed a mixture of underestimation and overestimation in specific samples, which proved reliable upon replication.

## Discussion

Two important conclusions can be drawn from these data: 1) The ability of indirect analytical tools to accurately capture levels of EDC metabolites cannot be assumed. Indeed, our data provide evidence of inaccuracy for all three chemicals analyzed, albeit with unique features for each. 2) Because biomonitoring data are key in estimating human exposure levels and assessing risk, accuracy, precision, sensitivity, and reproducibility are essential. Achieving this requires the use of the best analytical tools in the hands of a capable analytical chemist adhering to clinical guideline protocols [31].

### Indirect analytical tools: every EDC tells a different story

Methods that use validated metabolite standards provide more sensitive and accurate analytical tools than widely used indirect methods. Thus, when standards for the major metabolites of BPA, BPA-G and BPA-S became available, we developed direct methods for BPA measurement in maternal serum and urine samples [25, 32]. When direct analysis yielded significantly higher levels of BPA-G in some samples by comparison with indirect analysis, we examined the deconjugation reaction and found three problems; 1) a concentration-dependent diminution in the ability of the widely used *Helix pomatia* enzyme (extracted from snails) to deconjugate BPA-G, 2) that the reaction kinetics of the widely used surrogate substrate, 4-methylumbelliferone glucuronide, failed to model those of BPA-G, and 3) that deconjugation did not result in the expected concentration of the parent compound, BPA [26]. These findings not only raised concern about the accuracy of human BPA exposure estimates but, given the widespread use of indirect analytical methods for other EDCs, raised concern about the scope of the problem.

Undergraduate chemistry students learn that enzyme reaction kinetics are specific to the substrate and the enzyme acting upon it. Thus, it is remarkable that indirect analytical methods have relied on an assumption that defies this basic tenet: In the absence of standards for major EDC metabolites, metabolite levels in biospecimens have been inferred by comparing levels of the parent compound in samples before and after an enzymatic deconjugation reaction relying on hydrolysis by an enzyme extracted from snails. The kinetics of the deconjugation reaction, however, have been defined by deconjugation of a surrogate chemical and based on predictions about deconjugation products. Thus, the accuracy of these “indirect” methods depends both on the degree to which reaction kinetics of the surrogate align with those of the chemical of interest and the accuracy of the predicted products of enzymatic deconjugation. In the case of BPA, both conspire to create measurement inaccuracy. The current data provide evidence of the inaccuracy of indirect methods for three additional EDCs but do not provide insight into the cause (ie, deconjugation efficiency or products of the reaction).

The differences we identified in the accuracy of indirect analysis for the measurement of metabolites were evident for chemical representatives of three distinct classes of EDCs: BPS, PrP, and MBP. Although overall indirect analysis consistently underestimated metabolites of all three analytes, the extent of underestimation and its comparison with the direct method varied for each (Fig. 2 and Table 2). The greatest overall underestimation was observed for BPS, but unlike BPA, the underestimation of BPS was consistent across all concentration levels. MBP demonstrated a smaller degree of underestimation—approximately two-fold compared with ten-fold for BPS—and the bias gradually increased with higher concentrations. These trends are reflected in the comparison of results obtained using direct vs indirect analysis for the respective analytes (Fig. 2).

PrP showed the most complex behavior. Based on quartile differences alone, its level of underestimation appeared comparable to MBP. However, the data in Fig. 2 revealed variability among urine samples from pregnant women, with underestimation in a few samples and overestimation in others. This suggests that enzyme activity with respect to PrP is strongly influenced by biological variation, rendering the indirect method particularly unpredictable for this analyte in urine collected from second-trimester pregnant women.

We confirmed this conclusion by repeating the indirect analyses on four underestimated and four overestimated samples (data not shown). Both the underestimation and overestimation were confirmed on these samples. The imprecision of each analysis was within a coefficient of variation of 10%. In combination with our previous findings for BPA [26] and the confirmation of those findings here, these data provide further evidence that the assumption implicit in indirect analysis (ie, that reaction kinetics of a surrogate chemical can accurately predict deconjugation of the chemical of interest) is not valid for some common EDC environmental contaminants. Indeed, the results for two common bisphenols, BPA and BPS, demonstrate remarkable differences between structurally similar chemical analogs. Given the marked differences in their pharmacologic properties [33, 34], our data suggest pharmacology is a more important consideration than structural similarity.

Thus, the key finding from these studies is that the ability of indirect methods to accurately estimate metabolite levels in human urine is neither predictable nor consistent. Indeed, two factors that cannot be controlled are at play: the effectiveness of the deconjugation reaction and the accuracy of predictions about the products it will yield.

The logical conclusion from our data is that the accuracy of indirect methods in capturing metabolite levels must be established separately for each enzyme-analyte combination. Further, differences between BPA and BPS demonstrate that optimal deconjugation conditions can differ even for structurally similar analytes. Thus, applying the same reaction parameters to related analytes, as is currently the practice of the CDC and other laboratories, is scientifically unsound. Importantly, these concerns are alleviated by the use of validated standards to **directly** measure metabolites.

### Analytical chemists must adhere to clinical guideline protocols

Our findings demonstrate that accuracy in the analysis of biospecimens for EDCs requires adherence to protocols followed in clinical laboratories that include: (1) proficiency testing, (2) controlling and testing for contamination, and (3) monitoring the quality, accuracy and/or performance of standards and consumables.

The variability between the two analytical laboratories initially involved in our study demonstrates the difficulties inherent in the analysis of human biospecimens and the importance of proficiency testing. It is commonly assumed that analytical laboratories with methods for the analysis of EDCs are equally capable of analyzing human biospecimens. The two analytical laboratories are strikingly different: One is an academic laboratory that provides training for undergraduate and graduate students, while the other is a laboratory of analysts trained to conduct research under federal contracts. Only the latter was able to demonstrate sufficient proficiency to undertake subsequent analyses. Neither concordance among analytical laboratories nor the competence of a given analytical laboratory can be assumed in the absence of demonstrated proficiency for the required methods [35].

The inclusion of reference blanks and testing of collection and processing materials is our standard practice. Monitoring of commercially purchased standards revealed a lot with a misdeclared concentration. By running methods in parallel with currently used and new reagents (in this case, both the old and new standard) during lot changes, we were able to identify this discrepancy and prevent the generation of inaccurate data. Similarly, routine performance quality checks on the solid phase extraction cartridges (SPE) used in sample preparation identified a cartridge lot that failed to deliver reproducible results for one analyte (BPS-S), although performance for other analytes remained acceptable. Correction of this issue required waiting for six months for the vendor to release a replacement lot, highlighting the extent to which reagents and the quality of consumables can directly affect data integrity. Collectively, these findings underscore the numerous challenges inherent in generating reliable biomonitoring data. End users of analytical results must be aware and remain mindful of these challenges when engaging with an analytical laboratory to obtain exposure measurements.

## Summary and final conclusions

Estimates of human exposure are critical for risk assessments that guide the regulatory process. Thus, data from our limited comparisons of results using direct and indirect analytical tools underscore the importance of two considerations: First, to obtain the best exposure estimates, the data used must be obtained using the most accurate, reliable methods available. Second, because the greatest accuracy can be achieved using direct methods with validated standards for the major metabolites, it is essential that manufacturers and government agencies work to ensure that availability of standards keeps pace with the entry of new chemicals into the marketplace. For BPA, standards for metabolites only became available when NIEHS paid to have them synthesized for use in an NIEHS-sponsored round-robin validation study [35].

Changes necessary to enhance regulatory procedures and ensure protection of both human and environmental health have been the subject of extensive analysis and debate. Our findings, however, suggest that a simple change that would be neither onerous nor expensive, would have enormous impact: Prior to the introduction of a new chemical, manufacturers should be required to produce, validate, and make commercially available standards for the chemical and its major metabolites. This would be a minor expense in the process of developing and marketing chemicals for widespread use in products.

Clinicians, epidemiologists and biologists assume that biospecimens placed into the hands of a capable analytical chemist will yield reliable data on levels of environmental contaminants. Our data challenge this assumption and provide important insight to the difficulties faced by analytical chemists trying to measure EDC levels in human urine. Specifically: (1) Contamination, a persistent problem that shows up in surprising places, is an ongoing concern, (2) widely used indirect methods of estimating levels of metabolites are neither sufficiently reliable nor sensitive for the task at hand. Given the importance of biomonitoring in the estimation of human exposure levels, the development and adoption of direct analytical tools for measuring the EDCs that contaminate our environment is an essential step in safeguarding human health.

## Data Availability

Original data generated and analyzed during this study are included in this published article or in the data repositories listed in References.
